# Venous Thromboembolism and Pregnancy: Prevention and Treatment

**DOI:** 10.3390/jcm15218546

**Published:** 2026-11-03

**Authors:** Nneka Nwachukwu, Manila Gaddh

**Affiliations:** Division of Hematology, Department of Hematology and Medical Oncology, Emory University School of Medicine, Atlanta, GA 30322, USA

**Keywords:** thrombosis, pregnancy, postpartum, deep vein thrombosis, pulmonary embolism, low-molecular-weight heparin, VTE

## Abstract

Pregnancy-related venous thromboembolism (VTE) remains a leading cause of maternal morbidity and mortality. Physiologic changes in pregnancy, along with patient-specific and pregnancy-specific risk factors, increase this VTE risk. The prevention and treatment of pregnancy-related VTE is often complex and requires the careful consideration of variation in clinical guidelines, VTE risk profile, bleeding assessment, careful delivery planning and patient preference. The mainstay of treatment for an acute VTE in pregnancy is the use of low-molecular-weight heparin. In this review, we highlight three clinical cases to review clinical approaches to the prevention and treatment of VTE in pregnancy in these scenarios. Importantly, this review goes over the current understanding of the pathophysiology and clinical practice experience for pregnant patients with VTE. We also explored the variation in professional clinical guidelines on VTE prophylaxis and treatment strategies in pregnancy, and how these guidelines may be interpreted in the context of patient care for optimal outcomes.

## 1. Introduction

### 1.1. Background and Epidemiology

Venous thromboembolism (VTE), including deep vein thrombosis (DVT) and pulmonary embolism (PE), remains one of the leading causes of maternal morbidity and mortality. Compared to non-pregnant women of similar age, pregnant and postpartum women face a five- to ten-fold increased risk of VTE [1,2] with an estimated incidence worldwide ranging from 0.5 to 2.0 per 1000 pregnancies and a mortality rate of about one per 100,000 deliveries [2,3]. Most recent Centers for Disease Control and Prevention’s (CDC) Pregnancy Mortality Surveillance System data indicates that thrombotic pulmonary embolism accounted for ~11% of pregnancy-related deaths in the United States in 2024 [4]. According to an audit report from the UK and Ireland, VTE remained the leading cause of death from 2020 to 2022, accounting for 16% of pregnancy-related death [5]. A cohort study looking at pregnancy-associated VTE in Sweden from 1987 to 2019 reported 150.8 cases per 100,000 deliveries comprising 101.5 cases of DVT and 49.3 cases of PE [6]. Studies have shown conflicting data on the temporal trends in pregnancy-related VTE, with a recent study from Sweden showing an increasing trend in the incidence of pregnancy-associated PE over a 33-year period [6], in alignment with a large study conducted in the United States examining cases from 2000 to 2018 [7]. In contrast, a recent study out of the United States showed unchanged rates of acute PE during pregnancy between 2007 and 2015 [8].

This risk of VTE increases with each trimester, with the highest risk during the third trimester and the first six weeks after delivery [9]. This risk is even higher in patients with additional risk factors, such as prior VTE history, advanced maternal age, obesity, parity, thrombophilia (acquired or inherited), medical comorbidities such as diabetes, autoimmune condition and hypertension, and the use of assisted reproductive technology. Racial and ethnic disparities have also been associated with the incidence of VTE during pregnancy, with African American women having a higher risk than White Caucasian women [10].

### 1.2. Pathophysiology

The pathophysiology of VTE risk during pregnancy and postpartum is pathognomonic of the three elements of Virchow’s triad: hypercoagulability, venous stasis, and endothelial injury. In addition to these, more recent research has pointed to the role of epigenetic mechanisms in regulating the coagulation system during pregnancy, including impacting the coagulation cascade, angiogenesis, inflammation and immune responses, and, thus, further driving an increased risk of VTE [11,12].

a. Hypercoagulability—Pregnancy is associated with physiological changes in the coagulation and fibrinolytic systems, which are protective mechanisms to mitigate intrapartum blood loss, but may inadvertently lead to increased thrombotic risk. Key changes include.

- Increase in procoagulant factors (I, VII, VIII, X, XII) and von Willebrand factor (vWF);
- Acquired resistance to endogenous anticoagulants demonstrated by decreased protein S activity and acquired resistance to activated protein C [13];
- Decreased fibrinolytic activity in pregnancy due to an increase in the plasminogen activator inhibitor-1 (PAI-1) and plasminogen activator inhibitor type 2 (PAI-2), along with a decrease in tissue-type plasminogen activator (t-PA) activity [14].

Estrogen and progesterone, both of which are increased early in pregnancy, also play a key role in the pro-thrombotic state by increasing the synthesis of procoagulant factors, decreasing natural anticoagulants, as well as reducing fibrinolytic activity. Furthermore, changes in platelet activity further increase the risk of thrombosis. During pregnancy, there is an increased platelet reactivity to agonists such as the adenosine 5′ diphosphate, lowering the threshold for primary hemostasis [15].

b. Venous stasis—Pregnancy promotes venous stasis through mechanisms including hormonal changes, anatomic compression, and reduced mobility. The alterations in estrogen and progesterone hormonal levels not only induce a procoagulant state but, together with increased levels of relaxin during pregnancy, also promote venous stasis by systemic vasodilatation and increased venous capacitance [16]. As pregnancy progresses, the uterine enlargement causes direct anatomic compression to the left iliac vein analogous to May–Thurner anatomy. This results in an increased propensity for left-sided proximal vein (iliac and femoral) DVT with pregnancy [17,18]. Decreased mobility in pregnancy further exacerbates venous stasis and can be severe especially in pregnancy-related complications that require bed rest.

c. Endothelial damage—This may arise from damage to the pelvic veins which can be seen from venous hypertension or from increased vascular pressures during labor and delivery. The increased VTE risk seen postpartum is, in part, attributable to endothelial injury sustained during delivery, especially associated with cesarean delivery. This risk can be heightened in pre-eclampsia where there is a further increased endothelial damage.

### 1.3. Current Management Goals and Strategies

Beyond the short-term consequences related to VTE including increased bleeding risk associated with anticoagulation use and associated acute mortality, pregnancy-related VTE contributes to long-term complications such as post-thrombotic syndrome [19], poor quality of life [20], chronic thromboembolic pulmonary hypertension, as well as overall economic burden to the pregnant women and healthcare systems. Related to this, current recommended management goals aim to extend anticoagulation into the postpartum period, while mitigating maternal/fetal harm, along with careful delivery and postpartum planning through a multidisciplinary approach involving the hematologist, anesthesia, maternal–fetal medicine (MFM) and obstetrician/gynecologist (Ob/Gyn).

This purpose of this review was to summarize the current understanding of the prevention and treatment of VTE in pregnancy, utilizing practical, illustrative case-based discussions of common clinical scenarios encountered by the hematologist in the antepartum, peripartum and postpartum care periods.

## 2. Clinical Case Discussion

### 2.1. Clinical Case 1

A 32-year-old G1P0 woman at 12 weeks’ gestation presented to the emergency room with worsening left leg swelling. She denied any other symptoms including shortness of breath, chest pain, or leg pain. Her pregnancy had been unremarkable otherwise. She had no reported personal or family history of VTE. Physical examination was notable for a 2+ pitting edema isolated to her left ankle. A lower extremity Doppler ultrasound revealed an acute left femoral vein DVT.

#### 2.1.1. Pharmacologic and Non-Pharmacologic Management of VTE in Pregnancy

When VTE is diagnosed, anticoagulation therapy is the mainstay of management with low-molecular-weight heparin (LMWH) and unfractionated heparin (UFH) as key treatment options. The treatment of VTE in pregnancy is particularly challenging, as the safety of anticoagulant drugs and their potential adverse effects on the fetus, as well as the mother, must be considered carefully. Therapeutic-dose LMWH is the recommended treatment for VTE in pregnant patients as it does not cross the placenta and has a better safety profile compared to UFH [3,21,22]. Adverse effects of LMWH include injection site bruising, bleeding and the risk of heparin-induced thrombocytopenia (HIT) [21]. Commonly used LMWH includes enoxaparin, dalteparin and tinzaparin. Vitamin K antagonists (such as warfarin) cross the placenta and are not recommended in pregnancy due to well-established increased risks of teratogenicity [23], miscarriage and fetal bleeding. Fondaparinux and direct oral anticoagulants (DOACs) can also cross the placenta and have limited safety information on use in pregnancy [24,25,26]. However fondaparinux can be considered as an alternative option in pregnant women with HIT [27,28], heparin allergy [28] or LMWH failure. Systemic thrombolysis is reserved for pregnant patients with PE alongside life-threatening hemodynamic instability or cardiac arrest due to its associated increased bleeding risk [3,29].

The dosing strategies for LMWH use in pregnancy have generally been adopted from those used in the non-pregnant population. However, pregnancy is associated with physiologic changes including expanded plasma volume, increased renal clearance and increased body weight, all of which may alter LMWH pharmacokinetics, and have led to controversy and practice variation regarding the optimal dosing strategies of therapeutic LMWH in pregnancy—once vs. twice daily as well as the need for routine LMWH anti-Xa level monitoring. A small prospective multicenter observational study in the UK with 126 women receiving therapeutic antenatal LMWH, which employed various regimen (once vs. twice daily LMWH and anti-Xa monitoring), showed no difference in recurrent VTE outcome [30]. Similarly, a pharmacokinetics study by Patel et al. reported results suggesting the same [31]. In addition, another systematic literature review in Denmark showed limited data to support the benefits of routine anti-Xa monitoring in pregnant woman on LMWH therapy [32].

Several clinical guidelines suggest the use of actual weight-based LMWH dosing approach for the management of VTE during pregnancy over routine anti-factor Xa monitoring. Of note, differences have been noted in the pharmacokinetics of LMWH in patients with obesity compared to normal-weight patient, with obesity altering the clearance and volume of distribution of LMWH. There remain inadequate knowledge and evidence to guide optimal anticoagulation dosing in obese pregnant patients with VTE with dosing strategies and recommendations including traditional weight-based dosing and/or routine peak anti-Xa monitoring except in special circumstances, like extreme body weight and renal insufficiency [33]. A challenge remains that there is no established peak or trough anti-xa range for pregnant women to better correlate bleeding or VTE risk outcome [21,34]. Table 1 below summarizes the recommendations from the American Society of Hematology (ASH) [3], American College of Chest Physicians (ACCP) [35], and American College of Obstetricians and Gynecologists (ACOG) [36]. The recommended duration of anticoagulation therapy is for a minimum of 3 months and maintained throughout pregnancy and for at least 6 weeks postpartum. Therapeutic LMWH can be safely used with a minimal platelet count of 50 × 10^9^/L and in the absence of bleeding. If concurrent thrombocytopenia is present, additional workup will be needed to manage the disease process and safely continue therapeutic LMWH.

To balance the potential bleeding risk associated with delivery, the recommendation is to schedule delivery and hold therapeutic LMWH at least 24 h before induction of labor or cesarean section to allow time for neuraxial anesthesia. In case of spontaneous labor, LMWH should be held at the onset of labor and the use of neuraxial anesthesia should be avoided [3,21,22,37]. Transition to therapeutic UFH can be considered in patients with high risk of recurrent VTE (i.e., those with more recent thrombotic event within 2 weeks of expected delivery), which can be held 4–6 h before planned delivery or epidural placement. Postpartum, anticoagulation should be restarted once it is safe to do so and when hemostasis is achieved; this is generally 4 to 6 h after vaginal delivery or 6–12 h after cesarean delivery. The PRospective Evaluation of Peripartum Anticoagulation ManaGement for ThrOmboembolism (PREP and GO) study is an ongoing multicenter international prospective study evaluating the strategies for AC management around labor and delivery.

Non-pharmacological interventions include the use of compression stockings to alleviate leg symptoms. Retrievable Inferior Vena Cava (IVC) filter placement is generally avoided, especially in early fetal development due to radiation exposure. However, it can be safely considered for those with contraindications to or ineffective anticoagulation with its complication rate comparable to non-pregnant population [38].

#### 2.1.2. Back to Clinical Case 1

The patient was started on therapeutic enoxaparin 1 mg/kg subcutaneously every 12 h in the emergency department and continued throughout pregnancy. Hematology reviewed the limited data on routine anti-Xa monitoring and the ASH recommendation against routine anti-xa monitoring, which aligns with our department practice. However, her obstetric team elected to obtain monthly peak anti-Xa peak levels, which remained within target range throughout pregnancy and did not require dose adjustment. She was closely monitored by the obstetric team with monthly visits during her first two trimesters and every 2 weeks visit during her third trimester. She desired neuraxial anesthesia and opted to be electively induced at 39 weeks. Her enoxaparin was held 24 h prior to this scheduled delivery. She had an uncomplicated vaginal delivery, and enoxaparin was restarted 24 h post-delivery and continued for 6 weeks postpartum. She was counseled on the need for thromboprophylaxis with future pregnancies and avoidance of estrogen-containing contraception.

### 2.2. Clinical Case 2

A 30-year-old G0P0 woman with no significant past medical history was referred to hematology for preconception counseling regarding thromboprophylaxis during pregnancy. She has a known family history of protein C deficiency and unprovoked VTE in her father. She was otherwise healthy, with no personal history of VTE or additional VTE risk factors.

#### 2.2.1. VTE Risk Factors in Pregnancy

**Figure 1:**
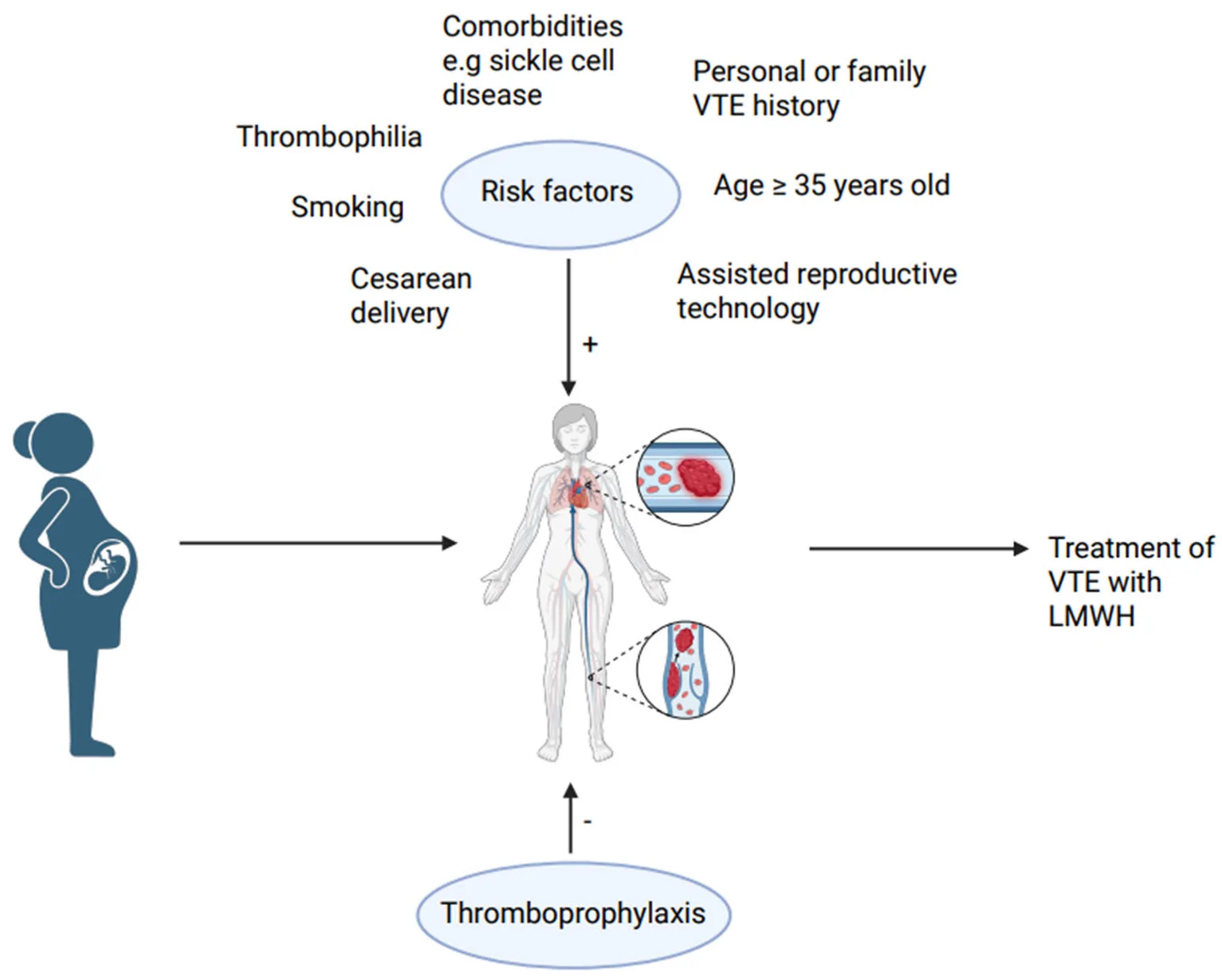
VTE in pregnancy. Created in BioRender. Nwachukwu, N. (2026) https://BioRender.com/i102lha.

In addition to the normal physiological changes in pregnancy increasing coagulability risk, additional factors such as inherited or acquired thrombophilia, as well as patient-specific and pregnancy-specific factors increase the risk further (Table 2 and Figure 1) with personal history of thrombosis carrying the highest risk. Importantly, these risk factors have been demonstrated to be additive for VTE in pregnancy [2]. These have also been demonstrated in validated risk scores for VTE risk in pregnancy [39]. Inherited thrombophilias include mutations affecting genes in the coagulation cascade such as protein C, protein S and antithrombin deficiencies, factor V Leiden and prothrombin G20210A gene mutations. While diagnosing thrombophilia during pregnancy can be challenging due to the physiological pregnancy-related hemostatic changes, identifying patients with thrombophilia is important as it informs anticoagulation management during pregnancy. Major guidelines like ASH and ACOG recommend the consideration of inherited thrombophilia screening in patients with first-degree relative history of high-risk inherited thrombophilia-antithrombin deficiency, homozygous factor V Leiden, homozygous PT gene mutation or compound heterozygosity [3,40]. However, testing should not be done during pregnancy, during an acute VTE, while on anticoagulation or hormonal therapy, as these can affect results. While commonly checked, there is insufficient evidence to suggest checking methylenetetrahydrofolate reductase (MTHFR) polymorphisms and homocysteine levels as part of the thrombophilia testing [40].

#### 2.2.2. What Are the Indications for Thromboprophylaxis in Pregnancy?

Despite the established increased VTE risk during pregnancy, there is no well-established consensus recommendation for thromboprophylaxis. Importantly, every clinical decision should ultimately be guided based on individual VTE risk–benefit assessment, family history of VTE, bleeding risk, and the preference of each pregnant individual in a shared decision-making and individual counseling. Thromboprophylaxis is generally indicated antepartum in pregnant patients with prior unprovoked or hormone-associated VTE and postpartum for 6 weeks in those with any prior VTE. However, there is variation in clinical guideline recommendations regarding thromboprophylaxis in pregnant women with inherited thrombophilias and no prior VTE, due insufficient high-quality data to inform decision. Table 3 summarizes the differences amongst the ACCP, ACOG and ASH recommendations for thromboprophylaxis use in individuals with inherited thrombophilias.

Optimal dosing for thromboprophylaxis varies with different professional society guidelines. For example, ACCP 2012 and ACOG 2018 suggest the use of either prophylactic or intermediate-dose LMWH; however, the ASH 2018 guidelines state preference for the use of prophylactic dose antepartum and either prophylactic or intermediate-dose postpartum. This question was further addressed in the Highlow study that looked at thromboprophylaxis with fixed low-dose (i.e., enoxaparin 40 mg daily for body weight < 100 kg) versus weight-adjusted intermittent dose of LMWH (i.e., enoxaparin 0.5 mg/kg every 12 h) [43]. This multicenter, open-labeled randomized controlled trial found no benefit with the use of weight-adjusted intermediate-dose LMWH when compared to fixed-dose prophylaxis in pregnant patients with a history of provoked (hormonal or minor-risk factors) or unprovoked VTE during pregnancy; however, post hoc analysis suggests better efficacy of intermediate-dose LMWH during the postpartum period.

#### 2.2.3. Assisted Reproductive Technology (ART)

Assisted reproductive technology includes a range of medical procedures to assist with human fertilization and pregnancy, involving handling both eggs and sperm outside the human body, with in vitro fertilization (IVF) being the most common. The risk of VTE in women undergoing assisted reproduction is two- to three-fold higher than in the general population [44], and more so in patients that develop ovarian hyperstimulation where the risk is increased 100-fold [45]. Ovarian hyperstimulation is associated with a hyperestrogenic state, thus increasing the VTE risk. For women undergoing assisted reproduction, the ACCP 2012 and ASH 2018 guidelines recommend against the use of routine thromboprophylaxis, except in the women who develop severe ovarian hyperstimulation syndrome [2,34].

Thromboprophylaxis is recommended for 3 months in patients with ovarian hyperstimulation post-resolution of the clinical syndrome [3,34]. While not addressed in most clinical guidelines due to insufficient evidence, some expert panels suggest the consideration of thromboprophylaxis in patients undergoing ART with previous VTE history or high-risk thrombophilia, if not already on long-term anticoagulation [3,46].

#### 2.2.4. Back to Clinical Case 2

In our practice, we typically follow the ASH guidelines while acknowledging the variation in professional society recommendations and ultimately shared decision-making with the patient. For this patient, given her first-degree family history of VTE and protein C deficiency, we recommended postpartum pharmacologic thromboprophylaxis with enoxaparin 40 mg daily for 6 weeks. She was also counseled on the signs and symptoms of VTE and associated increased VTE risk with estrogen-containing hormonal contraception.

### 2.3. Clinical Case 3

A 36-year-old G2P1 woman with hemoglobin SS sickle cell disease (SCD), who was 12-week pregnant at presentation, was referred to hematology for peripartum anticoagulation recommendation. The patient had a history of right common femoral DVT and PE during a previous hospitalization for viral infection and vaso-occlusive crisis one year prior, for which she was treated with a 3-month duration of anticoagulation for a presumed provoked event. Once pregnancy was confirmed, she was placed on prophylaxis enoxaparin (40 mg daily) by her primary care doctor but subsequently met with her MFM specialist who advised intermittent dose enoxaparin (40 mg every 12 h). She was otherwise healthy, with a prior uncomplicated pregnancy, infrequent hospitalizations and no reported SCD-related end-organ complications.

#### 2.3.1. VTE Management in Sickle Cell Disease

With advances in care for SCD, a growing number of individuals with SCD survive into reproductive years. However, the maternal and fetal morbidity and mortality remain high with estimates of up to 10- and 26-times higher mortality in SCD pregnancy vs. black and non-black controls, respectively [47], underscoring the need for specialized-multidisciplinary care during pregnancy.

Pregnancy and SCD both individually increase VTE risk. SCD is identified as a thrombophilic state, mediated by chronic activation of the coagulation system, the presence of chronic inflammation, endothelial disruption leading to the surface expression of adhesion molecules and increased platelet activation [48,49,50].

Pregnancy further exacerbates this VTE risk in SCD. Studies report an increased VTE risk in SCD pregnant women compared to non-SCD pregnant women [38,51], with the VTE rates of 2.8% vs. 0.05–0.2%, respectively, reported by Seaman et al. [52] and an approximate eight-fold increased PE risk has also been reported in a systematic review by Inparaj et al. [53] The VTE incidence in SCD patients may be comparable to that seen with high-risk inherited thrombophilias such as protein C and protein S deficiencies [48]. This VTE risk is also higher in those with prior VTE history, hydroxyurea use before pregnancy, as well as intrapartum painful crises and acute chest syndrome during pregnancy [54]. Importantly, SCD also carries an increased risk for postpartum hemorrhage (PPH), which is a major cause of maternal morbidity [55]. Early et al. reported an approximate two-fold increase in PPH in SCD pregnant women when compared to non-SCD black patients [47].

Despite this, there remains limited SCD-specific data to inform anticoagulation therapy during pregnancy; however, guidelines seem to agree that patients with additional risk factors should be considered for pharmacologic thromboprophylaxis. Care for VTE in SCD pregnant women is not addressed in the ASH 2018 guidelines on the management of VTE in pregnancy, although ASH recommends thromboprophylaxis in pregnant patients with history of previous VTE. The British SCD pregnancy guidelines recommend the consideration of pharmacologic thromboprophylaxis from 28 weeks through 6 weeks postpartum in individuals with SCD and earlier than 28 weeks if additional risk factors are present [56,57]. The World Health Organization (WHO) guidance published in 2025 recommends considering thromboprophylaxis in non-hospitalized SCD pregnant women with additional VTE risk factors [58]. While AC is beneficial, caution is needed to balance out the risk of postpartum hemorrhage which can be exacerbated with anticoagulation use. Prospective studies are therefore needed to further evaluate/define the benefits and harms of anticoagulation in this population.

#### 2.3.2. Back to Clinical Case 3

Given her history of VTE and her SCD, the hematology team reviewed her increased risk for VTE and discussed with the patient the option of thromboprophylaxis (enoxaparin 40 mg daily) through pregnancy. However, in a shared decision, the patient preferred to continue enoxaparin 40 mg every 12 h (versus switching to daily), which she remained on throughout her pregnancy and 6-week postpartum, including holding prior to delivery. At 8 weeks postpartum, in the absence of any symptoms and new risk factors for VTE, anticoagulation was discontinued safely. She was counseled against the use of estrogen-containing contraception given her increased risk for recurrent VTE.

## 3. Conclusions

Pregnancy-related VTE contributes to maternal and fetal morbidity and mortality. The prevention and treatment of VTE in the obstetric population remains a challenge due to varied clinical guideline recommendations, which are often based on small studies or extrapolated from non-pregnant populations. These clinical cases and discussions highlight some of the common clinical challenges faced considering limited guidelines available for the management of pregnancy-related VTE. It further highlights the need for high-quality research in this space to improve clinical management and outcomes in this population. The ongoing collaboration between ASH and the International Society on Thrombosis and Haemostasis (ISTH) aims to update the practice guidelines for the management of VTE in pregnancy. Prospective well-designed studies are needed to strengthen evidence and better inform clinician guidelines to optimize VTE prevention and treatment during pregnancy.

## Figures and Tables

**Table 1:** Recommendations of LMWH dosing regimen for VTE treatment in pregnancy.

Regimen	ACCP 2012 [35]	ACOG 2018 [36]	ASH 2018 [3]
Once vs. twice daily LMWH	No recommendation but notes that either dose regimen can be used	Prefers twice daily dosing but notes once daily as a possible alternative	Either
Weight-adjusted vs. routine anti-xa monitoring	- Favors weight-based dosing - No recommendation on routine anti-xa monitoring	No clear recommendation	- Favors weight-based dosing - Suggest against routine anti-Xa monitoring

**Table 2:** VTE risk factors during pregnancy [41,42].

Group	Risk Factors
Patient specific	Smoking Age (≥35 years of age) Medical comorbidities—i.e., lupus, sickle cell disease, obesity (BMI > 25 kg/m^2^), hypertension, diabetes and heart disease Previous VTE history—unprovoked or provoked (estrogen related) Family history of VTE (particularly first-degree relative)
Thrombophilia	Inherited thrombophilia—factor V Leiden, prothrombin G20210A gene mutation, antithrombin III, protein C and protein S deficiencies Acquired thrombophilia
Pregnancy specific	Pre-eclampsia or eclampsia Cesarean delivery Antepartum immobilization Postpartum infection Hemorrhage (ante and postpartum) Assisted reproductive technology Parity Multiple pregnancies

**Table 3:** Indication for thromboprophylaxis in pregnant women with inherited thrombophilia.

	ACCP 2012 [35]		ACOG 2018 [36]		ASH 2018 [3]	
	Antepartum	Postpartum	Antepartum	Postpartum	Antepartum	Postpartum
Homozygous FVL	Yes, if positive family VTE history	Yes	Yes	Yes	Yes	Yes
Homozygous PGM	Yes, if positive family VTE history	Yes	Yes	Yes	- No, if no family history of VTE. - Favors, if family history of VTE	Yes
Combined thrombophilia	No	Yes, if family history of VTE	Yes	Yes	Yes	Yes
Antithrombin deficiency	No	Yes, if family history of VTE	Yes	Yes	Yes, if family history of VTE	Yes, if family history of VTE
Protein C or S deficiency	No	Yes, if family history of VTE	Either surveillance or thromboprophylaxis	Either surveillance or thromboprophylaxis *	No	Yes, if family history of VTE
Heterozygous FVL or PGM	No	Yes, if positive family VTE history	Either surveillance or thromboprophylaxis	Either surveillance or thromboprophylaxis *	No	No

FVL—factor V Leiden. PGM—Prothrombin Gene mutation. * If additional VTE risk factors—first-degree relative with history of thrombotic episode or other major thrombotic risk factors.

## Data Availability

No new data were created or analyzed in this study. Data sharing is not applicable to this article.
